# Assessment of the nociceptive response to the use of cannabidiol alone and in combination with meloxicam through infrared pupillometry in female dogs undergoing elective ovariohysterectomy

**DOI:** 10.3389/fvets.2024.1380022

**Published:** 2024-07-04

**Authors:** Alejandro Casas-Alvarado, Julio Martínez-Burnes, Ismael Hernández-Ávalos, Patricia Mora-Medina, Agatha Miranda-Cortés, Adriana Domínguez-Oliva, Daniel Mota-Rojas

**Affiliations:** ^1^Doctorado en Ciencias Biológicas y de la Salud, Universidad Autónoma Metropolitana, Mexico City, Mexico; ^2^Facultad de Medicina Veterinaria y Zootecnia, Universidad Autónoma de Tamaulipas, Ciudad Victoria, Mexico; ^3^Clinical Pharmacology and Veterinary Anesthesia, Biological Sciences Department, FESC, Universidad Nacional Autónoma de México, Cuautitlán, Mexico; ^4^Facultad de Estudios Superiores Cuautitlán, Universidad Nacional Autónoma de México, Cuautitlán Izcalli, Mexico; ^5^Neurophysiology of Pain, Behavior and Assessment of Welfare in Domestic Animals, DPAA, Universidad Autónoma Metropolitana, Mexico City, Mexico

**Keywords:** pain, pupillometry, dogs, nociception, CBD, meloxicam

## Abstract

The negative effects of pain are a constant concern in the surgical management of animals, leading to the search for new drugs or more effective analgesic protocols to control this negative emotion. This study aimed to evaluate the nociceptive response of cannabidiol (CBD) alone and in combination with meloxicam using infrared pupillometry in female dogs undergoing elective ovariohysterectomy (OVH) under isoflurane anesthesia. A total of 60 female dogs of different breeds were included. These dogs were randomly assigned to four study groups according to the treatment: Control Group (G_0_: *n* = 15) receiving saline solution; group premedicated with meloxicam at a dose of 0.2 mg Kg^−1^ IV (G_Melox_: *n* = 15). Postoperatively this drug was used at 0.1 mg Kg^−1^ IV every 24 h; the CBD-treated Group (G_CBD_: *n* = 15) at a dose of 2 mg Kg^−1^ orally in the preoperative. Postoperatively was administrated every 12 h; and the Group premedicated with the combination of meloxicam and CBD (G_Melox/CBD_: *n* = 15) Meloxicam at a dose of 0.2 mg Kg^−1^ IV preoperatively, and 0.1 mg Kg^−1^ IV during the postoperative. CBD at a dose of 2 mg Kg^−1^ orally in the preoperative, and every 12 h in the postoperative. Treatments were administered for 48 postoperative hours. After OVH, the pupillary neurologic index, pupillary size, minimum diameter (MIN), percentage change, constriction latency (Lat), constriction velocity, and maximum constriction velocity were recorded as pupillometric variables in both eyes during events (E): Baseline (30 min before drug administration), E_30 min_, E_1h_, E_2h_, E_3h_, E_4h_, E_8h_, E_12h_, E_24h_, and E_48h_. The Short-Form of the Glasgow Composite Measure Pain Scale (GCMPS-SF) was used to assess pain during the same events. Overall, it was observed that the pupillometric variables Size, MIN., and Lat. were significantly higher in G_0_ compared to the other groups during E_30 min_, E_1h_, and E_2h_ (*p* = 0.03), indicating greater pupil dilation in G_0_ animals. Additionally, no statistically significant differences were observed in GCMPS-SF between G_Melox_, G_CBD_, and G_Melox/CBD_ during the postoperative period (*p* > 0.05). In contrast, the scores were statistically different compared to G_0_ (*p* = 0.00001), where all animals in this group received rescue analgesia at 2 h post-surgery. According to pupillometry and scores on the GCMPS-SF scale, it was observed that monotherapy with cannabidiol provides a similar analgesic effect to meloxicam alone or in combination with cannabidiol to manage acute pain in dogs. Similarly, these findings suggest that infrared pupillometry could be a tool for recognizing acute pain in dogs.

## 1 Introduction

Pain has physiological and emotional/behavioral negative outcomes in animals (1, 2). Therefore, it is a bioethical duty for the veterinarian to acknowledge and alleviate the perception of pain in animals under their care (3–5).

Pain management in companion animals relies on the use of analgesics such as opioids, non-steroidal analgesics (NSAIDs), and local analgesics. These drugs can prevent or decrease pain perception by interrupting some steps in the nociceptive neurobiology (6, 7). Despite the effectiveness of these analgesic drugs in several species, some authors state limitations in their use due to errors in clinical pain recognition, lack of pharmacological knowledge, or the risk of adverse effects (8, 9). For instance, opioids may cause respiratory depression and vasodilation, while NSAIDs may lead to adverse effects such as anorexia, vomiting, diarrhea, and negative consequences on renal and platelet function (10, 11).

An alternative to conventional analgesic drugs to manage pain in companion animals is the use of phytocannabinoid extracts, including cannabidiol (CBD) (12–14). In veterinary medicine, CBD is used as phytocannabinoid extracts (e.g., Sativex and Bedrocan) (15, 16), or synthetic cannabinoids such as CBD or tetrahydrocannabinol (THC). These highly liposoluble molecules interact with cannabinoid (CB) receptors 1 y CB2 (17, 18). Agonisms to CB1 receptors inhibit cAMP synthesis, inducing ion reduction. Consequently, the release of excitatory neurotransmitters (e.g., histamine, serotonin, dopamine, and glutamate) by the Central Nervous System (CSN) is reduced (19). Moreover, agonism of CB2 receptors reduces the inflammatory response induced by pro-inflammatory cytokines (20). It has been proposed that CBD can be used in combination with other drugs such as opioids to potentiate the analgesic effect due to shared mechanisms of action, reducing the dosage and minimizing the side effects of opioids (21–23). For multimodal analgesia, combining NSAIDs and CBD helps to prevent pain perception due to the action of each drug in different steps of the nociceptive pathway. However, there are limited studies evaluating the combination of NSAIDs with CBD during the perioperative period, although some reports indicate the reduction of pain perception in an osteoarthritis model (12, 24, 25).

Pupillometry is considered among the novel technological tools implemented to assess pain in dogs. It is suggested as a technique comparable to traditional methods that reduce evaluator subjectivity by quantitatively measuring pupillary diameter (26). In human medicine, this tool has been shown to objectively recognize pain and assess the efficacy of analgesic protocols to reduce their adverse effects (27). In veterinary medicine, although limited studies have been performed, Mills et al. (28) evaluated pupillometry in 126 healthy dogs to establish the pupillometric reference values for this species, which could help to develop pupillometric indices for pain assessment. Therefore, this study aimed to assess the nociceptive response of CBD alone or in combination with meloxicam through pupillometry in female dogs undergoing elective ovariohysterectomy under isoflurane anesthesia. It was hypothesized that animals receiving CBD alone or in combination with meloxicam would exhibit a lower nociceptive response compared to the use of meloxicam alone.

## 2 Materials and methods

### 2.1 Ethical considerations

Before carrying out the study, informed consent was obtained from the animals’ owners, authorizing the procedures. All work was performed under Mexico’s Official Norm NOM-062-ZOO-1999 guidelines on the technical specifications for animal production, care, and ethical use in applied ethological studies. This project was approved by the Academic Committee of the Ph.D. Program of Biological and Health Sciences (number CBS.066.21). Additionally, this study was conducted following the ARRIVE guidelines and ethical guidelines for the use of animals in experimentation (29, 30). No phase of the study during the surgical procedure or variable collection caused injury, mutilation, or overhandling of the animals.

### 2.2 Experimental design

Female dogs (*n =* 60) were randomly assigned into four groups according to the treatment: Control group (G_0_: *n =* 15) where 1 mL of saline solution was administered IV; Group premedicated with meloxicam (Meloxivet 5 mg/1 mL, Norvet, Mexico) (G_Melox_: *n =* 15) at a dose of 0.2 mg Kg^−1^ IV, 30 min before surgery. In the postoperative period, meloxicam was administered at 0.1 mg Kg^−1^ every 24 h (31); Group treated with CBD (extract of CBD with 1,000 mg/ 30 mL) (G_CBD_: *n =* 15) at a dose of 2 mg Kg^−1^ PO every 12 h (12); and Group medicated with the combination of meloxicam (0.2 mg Kg^−1^ IV and 0.1 mg Kg^−1^ every 24 h in the postoperative) and CBD (extract of CBD with 1,000 mg/ 30 mL) (2 mg Kg^−1^ PO every 12 h) (G_Melox/CBD_: *n =* 15). All treatments were administered 30 min before the start of surgery and in the immediate postoperative period for 48 h.

Pupillometry and the Glasgow Composite Measure Pain Scale (GCMPS-SF) scores were evaluated in the following events: Basal, 1 h before medical instrumentation (E_Basal_). Postoperative evaluations were performed at 30 min. (E_30min_), 1 h (E_1h_)_,_ 2 h (E_2h_), 3 h (E_3h_), 4 h (E_4h_), 8 h (E_8h_), 12 h (E_12h_), 24 h (E_24h_), and 48 h (E_48h_) after surgery.

### 2.3 Animals

Sixty female dogs of different breeds were included in the present study (21 mixed breed, 9 Chihuahua, 8 Poodle, 7 Pitbull, 5 Schnauzer, 2 Bobtail, 2 Cocker Spaniel, 2 Beagle, 1 Shiba, 1 Golden Retriever, 1 Teckel, and 1 Siberian Husky). Dogs had an average age, body condition score, and body weight of 2 ± 1.5 years, 3/5, and 12.1 ± 2.3 kg, respectively. The sample size was estimated using G*power 3.1.9.7 software (Heinrich-Heine-Universität Düsseldorf, Düsseldorf, Alemania) (32). To determine the sample size for four experimental groups and 10 measurements, an α error of 0.05 was established, with a confidence level of 95%, power (1- α error probability) of 0.95, and a correction among repeated measures of 0.5 (33).

All animals enrolled in the study underwent preanesthetic evaluation through a comprehensive general physical examination and laboratory tests, including complete blood cell count, serum biochemistry, and urinalysis, performed 24 h before surgery. Clinically healthy animals meeting the criteria for an ASA1 anesthetic risk according to the American Society of Anesthesiologists (34) were selected. Patients with ASA2 or higher anesthetic risk were excluded. Brachycephalic breeds, dogs medicated with anticholinergics, and with other conditions causing acute pain, with serious infectious or ocular diseases that could interfere with pupillometric evaluation were also excluded.

### 2.4 Anesthesia and perioperative management

Elective ovariohysterectomy (OVH) was performed with the previous informed consent of the owner. Dogs had 6-h fasting for food and 4-h fasting for water before the surgical procedure.

Animals were aseptically catheterized in the cephalic vein with a number 20G intravenous catheter. Ringer lactate solution was administered at an infusion rate of 5 mL Kg^−1^ h^−1^ (BeneFusion VP1 Vet, Mindray, Germany) during the surgical procedure (35).

Once catheterized, the animals were premedicated with Dexmedetomidine (Dexdomitor 0.5 mg/ 1 mL, Zoetis, Mexico) at a dose of 1.5 μg Kg^−1^ intravenously (IV). Five minutes after premedication, the dogs presented moderate sedation according to Grint et al. (36)‘s sedation score. Anesthetic induction was performed with Propofol (Recofol 1%, Pisa, Mexico) at 2–4 mg Kg^−1^ IV (37). Once an adequate state of unconsciousness was observed (e.g., ventromedial deviation of the eyeball and decreased jaw tone), orotracheal intubation was performed. The orotracheal tube was connected to an anesthetic rebreathing circuit with an oxygen flow of 45 mL Kg^−1^ min^−1^. Anesthetic maintenance was performed with isoflurane (Sofloran, Pisa, Mexico) vaporized in 100% oxygen, regulating the vaporizer dial initially at 1.8% and modifying the concentration according to the anesthetic depth required to maintain a mean arterial pressure (MAP) between 60 to 90 mmHg, assessed through non-invasive blood pressure. All animals were ventilated with a mechanical ventilator into the anesthesia station (Wato-EX20 vet, Mindray, Germany), using a pressurometric ventilation method controlled at a mean airway pressure (Paw) of 10–15 cmH20 and an I:E ratio of 1:2 during surgery. A respiratory rate of 12 to 20 breaths per minute was established to maintain an EtCO2 of 35–45 mmHg (ePM12VETc/AA, Mindray, Alemania).

The surgical anesthetic depth was assessed through the recognition of clinical signs such as jaw tone relaxation, ventromedial deviation of the eyeball, and the absence of the palpebral reflex. All OVH surgeries were performed by the same surgeon using a midline approach and a triple hemostatic surgical technique. Similarly, all anesthetic procedures were carried out by the same anesthesiologist. The administration of inhalant anesthetics stopped 5 min before surgical wound closure. The end of the surgery was considered after the closure of the surgical incision. Extubating with the reappearance of the cough reflex was performed when patients could successfully sustain spontaneous ventilation and returned the ocular globe to the central position.

### 2.5 Infrared pupillometry

**Figure 1:**
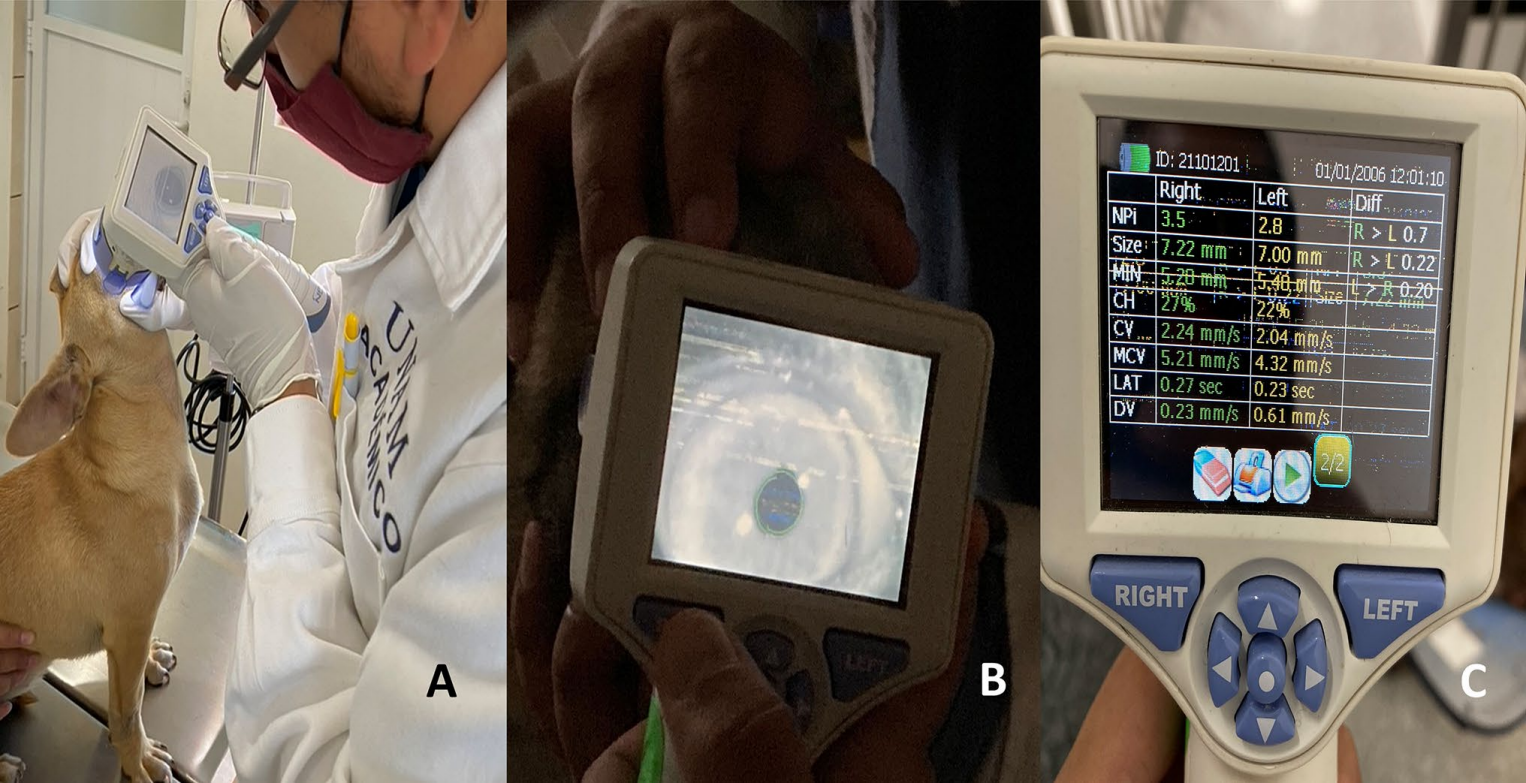
Methodology of the infrared pupilometry technique. **(A)** The placement of the pupilometer at a 90° angle in the ocular region is depicted. **(B)** The moment of measuring the pupil diameter using the infrared light camera is shown. From the pupil diameter measurement, 7 different variables are captured, including the neurological pupil index (NPi), size, minimum diameter (MIN), percentage change (% CH), constriction latency (LAT), constriction velocity (CV), and maximum constriction velocity (MCV), as shown in image **C**.

An automated and portable pupillometer (Neuroptics, NPi 200, United States) was used to measure pupillary size during 60 s in each eye (Figure 1). The following parameters were registered: neurological pupil index (NPi), size, minimum diameter (MIN), percentage of change (% CH), constriction latency (LAT), constriction velocity (CV) and maximum constriction velocity (MCV) (38). Pupillary assessment was performed once in each event. Assessments were performed by a single blinded evaluator.

### 2.6 Assessment of acute pain

The Glasgow Composite Pain Score – Short Form (GCMPS-SF) was used to assess pain. This scale comprises different behavioral and physiological categories, as well as response to touch, facial expression, vocalization, and mobility. The maximum pain score is 24 points (39). A single and trained evaluator performed all measures. Rescue analgesia with Tramadol (Tramajet 50 mg/ 1 mL; Norvet, Mexico) at 4 mg kg ^−1^ IV (40–42) was administered in the postsurgical period when GCMPS-SF score was ≥6 points.

### 2.7 Statistical analyses

Descriptive statistics were obtained using Graph Pad Prism (ver. 9.5) for all groups (G_0_, G_Melox_, G_CBD_, G_Melox/CBD_) and all events (E_Basal_, E_30 min_, E_1h_, E_2h_, E_3h_, E_4h_, E_8h_, E_12h_, E_24h_, and E_48h_). Normality tests were done with the Kolmogorov–Smirnov test for all the variables assessed.

The treatments were considered independent variables, while each of the pupillometric parameters and the post-surgical pain evaluation scores were considered dependent. To evaluate the effects of these variables, a linear mixed model was used.

A Tukey *post hoc* test was used to evaluate differences between means. The analysis of sensitivity and specificity was carried out using a receiver operating characteristics (ROC) test using the score obtained in GCMPS-SF as the gold standard. Finally, the linear relationship between study variables was performed using a Pearson correlation test. In all cases, the significance level was set at *p* < 0.05.

## 3 Results

In the present study, 64 dogs were considered. However, four dogs were excluded: two dogs due to the administration of anticholinergics, one dog due to pyometra, and one dog due to osteoarthritic chronic pain. A total of 60 dogs were included, 21 mixed breeds, 9 Chihuahuas, 8 Poodles, 7 Pitbulls, 5 Schnauzers, 2 Bobtail, 2 Cocker Spaniel, 2 Beagle, 1 Shiba, 1 Golden Retriever, 1 Teckel, and 1 Siberian Husky. In general, the average anesthesia time was 57 ± 8.4 min, surgical time was 24 ± 4.8 min, and extubating time was 13 ± 2.8 min. The main findings of the pupillary assessment show that Size, MIN, and Lat, had significant differences between groups (*p <* 0.05) particularly G_Melox_, G_CBD_, and G_Melox/CBD_ with G_0_. These differences were observed during the first two postoperative hours. Moreover, all animals in G_0_ required rescue analgesia at E_2h_.

In Table 1, it can be observed that the Size of the right eye (maximum pupil diameter) significantly increased in G_CBD_ during E_2h_ (*p =* 0.006) when comparing basal values in the same group, registering 9.19 ± 0.26 mm. During E_2h,_ the Size of G_Melox/CBD_ was 8.59 ± 0.30 mm, a value that was not statistically significant (*p =* 0.47) in comparison with G_CBD_ (9.19 ± 0.26 mm) and G_Melox_ (9.35 ± 0.20 mm). However, the pupil diameter of G_0_ was 9.90 ± 0.07 mm, showing statistically significant differences (*p =* 0.003) with the other experimental groups G_Melox_, G_CBD_, G_Melox/CBD_.

**Table 1:** Pupillometric values (Mean ± EE) of the right eye pupil in the evaluation events (E) of 60 bitches under elective ovariohysterectomy surgery distributed in 4 study groups: G_0_, G_Melox_, G_CBD_, G_Melox/CBD_.

Parameters	Treatments	Post-surgical Events										*p* value
		E_Basal_	E_30Min._	E_1h._	E_2h._	E_3h._	E_4h._	E_8h._	E_12h._	E_24h._	E_48h._	
NPi	G_0_ *n =* 15	4.2^1,a^ ± 0.18	4.3^1,a^ ± 0.20	4.3^1,a^ ± 0.20	4.2^1,a^ ± 0.16	4.3^1,a^ ± 0.18	4.3^1,a^ ± 0.17	4.2^1,a^ ± 0.19	4.6^1,a^ ± 0.08	4.3^1,a^ ± 0.16	4.4^1,a^ ± 0.20	*p* > 0.05
	G_Melox_, *n* = 15	3.9^1,a^ ± 0.25	4.3^1,a^ ± 0.15	4.2^1,a^ ± 0.15	4.2^1,a^ ± 0.14	4.2^1,a^ ± 0.15	4.2^1,a^ ± 0.15	4.5^1,a^ ± 0.14	4.2^1,a^ ± 0.21	4.2^1,a^ ± 0.20	4.4^1,a^ ± 0.14	*p* > 0.05
	G_CBD_ *n =* 15	4.1^1,a^ ± 0.21	4.3^1,a^ ± 0.13	4.1^1,a^ ± 0.14	4.2^1,a^ ± 0.14	4.5^1,a^ ± 0.09	4.3^1,a^ ± 0.14	4.4^1,a^ ± 0.10	4.4^1,a^ ± 0.14	3.8^1,a^ ± 0.13	4.2^1,a^ ± 0.12	*p* > 0.05
	G_Melox/CBD_ *n =* 15	4.4^1,a^ ± 0.11	4.2^1,a^ ± 0.14	4.3^1,a^ ± 0.13	4.3^1,a^ ± 0.11	4.4^1,a^ ± 0.14	4.5^1,a^ ± 0.08	4.3^1,a^ ± 0.12	4.5^1,a^ ± 0.11	4.0^1,a^ ± 0.14	4.5^1,a^ ± 0.08	*p* > 0.05
	*P* value	*p* > 0.05	*p* > 0.05	*p* > 0.05	*p* > 0.05	*p* > 0.05	*p* > 0.05	*p* > 0.05	*p* > 0.05	*p* > 0.05	*p* > 0.05	
Size (mm.)	G_0_ *n =* 15	9.01^1,a^ ± 0.42	9.53^1,a^ ± 0.18	9.46^1,a^ ± 0.16	9.90^1,a^ ± 0.07	9.73^1,a^ ± 0.15	9.73^1,a^ ± 0.15	9.04^1,a^ ± 0.27	9.88^1,a^ ± 0.07	9.39^1,a^ ± 0.16	9.58^1,a^ ± 0.19	*p* > 0.05
	G_Melox_, *n* = 15	8.52^1,a^ ± 0.42	9.54^1,a^ ± 0.15	9.52^1,a^ ± 0.19	9.35^2,a^ ± 0.20	9.54^1,a^ ± 0.22	9.47^1,a^ ± 0.23	9.54^1,a^ ± 0.15	9.77^1,a^ ± 0.18	8.94^1,a^ ± 0.50	8.96^1,a^ ± 0.29	*p* > 0.05
	G_CBD_ *n =* 15	8.17^1,b^ ± 0.49	9.02^1,a^ ± 0.21	8.96^1,a^ ± 0.24	9.19^2,a^ ± 0.26	9.06^1,a^ ± 0.16	9.52^1,a^ ± 0.20	9.60^1,a^ ± 0.13	9.58^1,a^ ± 0.20	9.66^1,a^ ± 0.21	9.58^1,a^ ± 0.23	***p* = 0.006**
	G_Melox/CBD_ *n =* 15	9.39^1,a^ ± 0.37	8.75^1,a^ ± 0.24	8.73^1,a^ ± 0.31	8.59^2,a^ ± 0.30	9.08^1,a^ ± 0.25	8.93^1,a^ ± 0.20	9.17^1,a^ ± 0.23	9.30^1,a^ ± 0.18	9.44^1,a^ ± 0.22	9.07^1,a^ ± 0.36	*p* > 0.05
	*P* value	*p* > 0.05	*p* > 0.05	*p* > 0.05	***p* = 0.003**	*p* > 0.05	*p* > 0.05	*p* > 0.05	*p* > 0.05	*p* > 0.05	*p* > 0.05	
MIN (mm.)	G_0_ *n =* 15	5.89^1,a^ ± 0.36	6.99^1,a^ ± 0.38	7.27^1,a^ ± 0.42	7.33^1,a^ ± 0.33	7.21^1,a^ ± 0.48	6.82^1,a^ ± 0.33	6.08^1,a^ ± 0.32	6.42^1,a^ ± 0.39	6.78^1,a^ ± 0.37	6.95^1,a^ ± 0.47	*p* > 0.05
	G_Melox_, *n* = 15	6.09^1,a^ ± 0.30	6.43^1,a^ ± 0.28	6.39^1,a^ ± 0.24	6.22^1,a^ ± 0.29	6.38^1,2,a^ ± 0.28	5.98^1,a^ ± 0.35	5.83^1,a^ ± 0.33	5.56^1,a^ ± 0.43	5.43^1,a^ ± 0.22	5.74^1,a^ ± 0.23	*p* > 0.05
	G_CBD_ *n =* 15	5.55^1,a^ ± 0.27	6.03^1,a^ ± 0.17	5.80^2,a^ ± 0.20	5.62^2,a^ ± 0.14	5.82^1,2,a^ ± 0.15	6.04^1,a^ ± 0.18	6.32^1,a^ ± 0.33	6.14^1,a^ ± 0.20	6.29^1,a^ ± 0.37	6.15^1,a^ ± 0.27	*p* > 0.05
	G_Melox/CBD_ *n =* 15	6.00^1,a^ ± 0.26	5.87^1,a^ ± 0.28	5.96^2,a^ ± 0.30	5.98^3,a^ ± 0.25	5.74^2,a^ ± 0.31	5.89^1,a^ ± 0.36	6.55^1,a^ ± 0.22	6.04^1,a^ 0.31	6.47^1,a^ ± 0.24	6.10^1,a^ ± 0.25	*p* > 0.05
	*P* value	*p* > 0.05	*p* > 0.05	***p* = 0.01**	***p* = 0.03**	***p* = 0.003**	*p* > 0.05	*p* > 0.05	*p* > 0.05	*p* > 0.05	*p* > 0.05	
CH (%)	G_0_ *n =* 15	28.40^1,a^ ± 2.19	32.29^1,a^ ± 2.70	36.13^1,a^ ± 2.76	31.50^1,a^ ± 2.14	33.88^1,a^ ± 2.08	33.22^1,a^ ± 1.57	31.20^1,a^ ± 2.17	33.75^1,a^ ± 2.44	33.00^1,a^ ± 2.38	32.25^1,a^ ± 3.50	*p* > 0.05
	G_Melox_, *n* = 15	28.89^1,a^ ± 3.22	33.36^1,a^ ± 2.19	29.62^1,a^ ± 1.73	29.85^1,a^ ± 1.75	29.09^1,a^ ± 1.47	31.82^1,a^ ± 2.12	31.60^1,a^ ± 2.63	32.27^1,a^ ± 2.31	31.55^1,a^ ± 2.96	35.56^1,a^ ± 2.45	*p* > 0.05
	G_CBD_ *n =* 15	27.82^1,a^ ± 1.40	30.50^1,a^ ± 1.75	29.31^1,a^ ± 1.20	31.54^1,a^ ± 1.49	35.43^1,a^ ± 1.65	33.13^1,a^ ± 2.01	31.79^1,a^ ± 1.91	34.45^1,a^ ± 2.11	27.71^1,a^ ± 1.33	30.43^1,a^ ± 1.28	*p* > 0.05
	G_Melox/CBD_ *n =* 15	28.85^1,a^ ± 2.33	32.17^1,a^ ± 1.93	31.42^1,a^ ± 1.23	33.85^1,a^ ± 1.78	34.00^1,a^ ± 2.48	33.64^1,a^ ± 2.85	30.92^1,a^ ± 1.66	35.09^1,a^ ± 2.59	28.42^1,a^ ± 1.14	33.36^1,a^ ± 1.79	*p* > 0.05
	*P* value	*p* > 0.05	*p* > 0.05	*p* > 0.05	*p* > 0.05	*p* > 0.05	*p* > 0.05	*p* > 0.05	*p* > 0.05	*p* > 0.05	*p* > 0.05	
CV (mm./seg.)	G_0_ *n =* 15	3.59^1,a^ ± 0.21	3.44^1,a^ ± 0.32	3.77^1,a^ ± 0.26	2.91^1,a^ ± 0.22	3.37^1,a^ ± 0.18	3.45^1,a^ ± 0.16	3.34^1,a^ ± 0.21	2.99^1,a^ ± 0.29	3.12^1,a^ ± 0.17	3.06^1,a^ ± 0.26	*p* > 0.05
	G_Melox_, *n* = 15	3.23^1,a^ ± 0.26	3.43^1,a^ ± 0.24	3.32^1,a^ ± 0.20	3.22^1,a^ ± 0.20	3.08^1,a^ ± 0.20	3.14^1,a^ ± 0.20	3.45^1,a^ ± 0.24	3.45^1,a^ ± 0.40	3.08^1,a^ ± 0.25	3.67^1,a^ ± 0.30	*p* > 0.05
	G_CBD_ *n =* 15	2.56^1,a^ ± 0.23	3.15^1,a^ ± 0.24	3.40^1,a^ ± 0.19	3.35^1,a^ ± 0.22	3.36^1,a^ ± 0.20	3.41^1,a^ ± 0.20	3.37^1,a^ ± 0.19	3.41^1,a^ ± 0.23	2.76^1,a^ ± 0.24	2.88^1,a^ ± 0.27	*p* > 0.05
	G_Melox/CBD_ *n =* 15	3.33^1,a^ ± 0.28	3.19^1,a^ ± 0.21	3.11^1,a^ ± 0.28	3.92^1,a^ ± 0.28	3.82^1,a^ ± 0.18	3.30^1,a^ ± 0.28	3.47^1,a^ ± 0.24	3.37^1,a^ ± 0.28	3.50^1,a^ ± 0.24	3.42^1,a^ ± 0.27	*p* > 0.05
	*P* value	*p* > 0.05	*p* > 0.05	*p* > 0.05	*p* > 0.05	*p* > 0.05	*p* > 0.05	*p* > 0.05	*p* > 0.05	*p* > 0.05	*p* > 0.05	
MCV (mm./seg.)	G_0_ *n =* 15	5.63^1,a^ ± 0.37	5.82^1,a^ ± 0.39	5.96^1,a^ ± 0.51	5.55^1,a^ ± 0.36	5.96^1,a^ ± 0.32	5.42^1,a^ ± 0.32	6.00^1,a^ ± 0.42	5.00^1,a^ ± 0.51	5.27^1,a^ ± 0.44	5.75^1,a^ ± 0.40	*p* > 0.05
	G_Melox_, *n* = 15	5.35^1,a^ ± 0.38	6.00^1,a^ ± 0.37	6.04^1,a^ ± 0.23	5.83^1,a^ ± 0.36	6.07^1,a^ ± 0.28	6.18^1,a^ ± 0.34	5.80^1,a^ ± 0.40	5.86^1,a^ ± 0.57	5.47^1,a^ ± 0.56	6.16^1,a^ ± 0.51	*p* > 0.05
	G_CBD_ *n =* 15	5.57^1,a^ ± 0.48	5.88^1,a^ ± 0.42	5.92^1,a^ ± 0.35	5.80^1,a^ ± 0.32	6.19^1,a^ ± 0.33	5.43^1,a^ ± 0.33	5.84^1,a^ ± 0.37	5.87^1,a^ ± 0.39	4.88^1,a^ ± 0.30	5.1^1,a^ ± 0.33	*p* > 0.05
	G_Melox/CBD_ *n =* 15	5.47^1,a^ ± 0.31	5.96^1,a^ ± 0.30	5.38^1,a^ ± 0.25	6.25^1,a^ ± 0.27	6.11^1,a^ ± 0.40	5.72^1,a^ ± 0.39	5.64^1,a^ ± 0.26	5.86^1,a^ ± 0.57	5.45^1,a^ ± 0.26	5.90^1,a^ ± 0.33	*p* > 0.05
	*P* value	*p* > 0.05	*p* > 0.05	*p* > 0.05	*p* > 0.05	*p* > 0.05	*p* > 0.05	*p* > 0.05	*p* > 0.05	*p* > 0.05	*p* > 0.05	
Lat (mm./seg.)	G_0_ *n =* 15	0.20^1,a^ ± 0.01	0.38^1,b^ ± 0.03	0.24^1,b^ ± 0.02	0.28^1,b^ ± 0.03	0.24^1,a^ ± 0.02	0.26^1,a^ ± 0.02	0.22^1,a^ ± 0.02	0.26^1,a^ ± 0.04	0.25^1,a^ ± 0.03	0.24^1,a^ ± 0.02	***p* = 0.0001**
	G_Melox_, *n* = 15	0.21^1,a^ ± 0.01	0.22^2,a^ ± 0.00	0.20^1,a^ ± 0.00	0.25^1,2,a^ ± 0.01	0.23^1,a^ ± 0.00	0.20^1,a^ ± 0.00	0.21^1,a^ ± 0.01	0.22^1,a^ ± 0.01	0.22^1,a^ ± 0.01	0.19^1,a^ ± 0.00	*p* > 0.05
	G_CBD_ *n =* 15	0.22^1,a^ ± 0.01	0.22^2,a^ 0.00	0.22^1,a^ ± 0.01	0.22^1,2,a^ ± 0.00	0.20^1,a^ ± 0.01	0.23^1,a^ ± 0.01	0.26^1,a^ ± 0.03	0.23^1,a^ ± 0.01	0.24^1,a^ ± 0.01	0.21^1,a^ ± 0.00	*p* > 0.05
	G_Melox/CBD_ *n =* 15	0.22^1,a^ ± 0.01	0.20^2,a^ ± 0.01	0.20^1,a^ ± 0.00	0.19^2,a^ ± 0.00	0.20^1,a^ ± 0.01	0.19^1,a^ ± 0.01	0.21^1,a^ ± 0.02	0.22^1,a^ ± 0.01	0.22^1,a^ ± 0.01	0.20^1,a^ 0.01	*p* > 0.05
	*P* value	*p* > 0.05	***p* = 0.003**	*p* > 0.05	***p* = 0.02**	*p* > 0.05	*p* > 0.05	*p* > 0.05	*p* > 0.05	*p* > 0.05	*p* > 0.05	

^a,b,c^Different literals by row indicate significant differences between events for the same treatment. ^1,2,3,4^Different numerals by column indicate significant differences between treatments for the same event. T = treatments (G_0_: negative group, G_Melox_: Meloxicam group, G_CBD_: Cannabidiol group, G_Melox/CBD_: Mexociam and Cannabidiol group). E: post-surgical events (E_Basal_: 30 min. Pre-surgery; E_30 min._: 30 min post- surgery; E_1h._: 1 h post-surgery; E_2h._: 2 h post-surgery; E_3h._: 3 h post-surgery; E_4h._: 4 h post-surgery; E_8h._: 8 h post-surgery; E_12h._: 12 h post-surgery, E_24h._: 24 h post-surgery; E_48h._: 48 h post-surgery). NPi, Neurological pupil index; Size, Maximum pupil size before constriction; MIN, Pupil diameter at peak constriction; CH, Percentage of pupil change. CV, Constriction velocity. MCV, Maximum constriction velocity. Lat., Latency of constriction. Bold values represent statistically significant differences.

In the case of the minimum pupil diameter (MIN) of the right eye, statistically significant differences were reported between study groups during E_1h,_ (*p =* 0.01), E_2h,_ (*p =* 0.03), and E_3h,_ (*p =* 0.003). Animals in G_0_ recorded the highest values with 7.27 ± 0.42, 7.33 ± 0.33, and 7.21 ± 0.48 mm at E_1h,_ E_2h,_ and E_3h,_ respectively.

Regarding the latency time of pupillary constriction (Lat), the Lat of G_0_ animals increased between 0.08 and 0.18 s compared to the rest of the postsurgical events and the E_Basal._ from the same experimental group (*p =* 0.0001). Likewise, at E_30min_ and E_2h,_ statistically significant differences between treatments were reported (*p =* 0.003 y *p =* 0.02 respectively). The latency time in G_0_ was 0.38 ± 0.03 s during E_30min,_ while at E_2h,_ Lat. was 0.28 ± 0.03. sec. In contrast, values recorded from G_Melox_, G_CBD_, G_Melox/CBD_ decreased between 0.16–0.18 and 0.03–0.09 s, respectively, during the evaluation events. Also in Table 1, it can be observed that NPi, CH, CV, and MCV did not have significant differences between treatments and/or events (*p* > 0.05).

Table 2 shows the pupillometric variables of the left eye. Similar to the previously described results, CH, CV, and MCV had no statistical differences between events or between treatments (*p >* 0.05). However, NPi values increased in the left eye (between 0.70–1.00) in all postsurgical events when compared to E_Basal.,_ where a value of 3.60 ± 0.24 (*p =* 0.03) was recorded. For the Size variable in the left eye, the diameter of animals in G_Melox_ at E_24h_ was significantly smaller compared to the rest of the events (*p =* 0.006) and between treatments (*p =* 0.002).

**Table 2:** Pupillometric values (Mean ± EE) of the left eye pupil in the evaluation events (E) of 60 bitches under elective ovariohysterectomy surgery distributed in 4 study groups: G_0_, G_Melox_, G_CBD_, G_Melox/CBD_.

Parameters	Treatments	Post-surgical events										*P* value
		E_Basal_	E_30Min._	E_1h._	E_2h._	E_3h._	E_4h._	E_8h._	E_12h._	E_24h._	E_48h._	
NPi	G_0_ *n =* 15	3.96^1,a^ ± 0.19	4.04^1,a^ ± 0.23	4.17^1,a^ ± 0.31	3.97^1,a^ ± 0.24	4.35^1,a^ ± 0.14	4.03^1,a^ ± 0.16	4.30^1,a^ ± 0.09	4.24^1,a^ ± 0.11	4.37^1,a^ ± 0.11	4.25^1,a^ ± 0.15	*p* > 0.05
	G_Melox_, *n* = 15	3.99^1,a^ ± 0.21	4.32^1,a^ ± 0.11	3.93^1,a^ ± 0.24	4.22^1,a^ ± 0.14	4.31^1,a^ ± 0.10	4.35^1,a^ ± 0.10	4.50^1,a^ ± 0.11	4.25^1,a^ ± 0.19	4.11^1,a^ ± 0.13	3.93^1,a^ ± 0.18	*p* > 0.05
	G_CBD_ *n =* 15	3.90^1,a^ ± 0.19	3.95^1,a^ ± 0.20	4.43^1,a^ ± 0.08	4.45^1,a^ ± 0.09	4.29^1,a^ ± 0.14	4.47^1,a^ ± 0.09	4.48^1,a^ ± 0.14	4.32^1,a^ ± 0.14	4.14^1,a^ ± 0.15	4.22^1,a^ ± 0.14	*p* > 0.05
	G_Melox/CBD_ *n =* 15	3.60^1,a^ ± 0.24	4.38^1,b^ ± 0.10	4.30^1,b^ ± 0.09	4.33^1,b^ ± 0.10	4.44^1,b^ ± 0.12	4.60^1,b^ ± 0.05	4.50^1,b^ ± 0.08	4.49^1,b^ ± 0.08	4.50^1,b^ ± 0.05	4.22^1,b^ ± 0.13	***p* = 0.03**
	*P* value	*p* > 0.05	*p* > 0.05	*p* > 0.05	*p* > 0.05	*p* > 0.05	*p* > 0.05	*p* > 0.05	*p* > 0.05	*p* > 0.05	*p* > 0.05	
Size (mm.)	G_0_ *n =* 15	8.90^1,a^ ± 0.29	9.57^1,a^ ± 0.21	9.65^1,a^ ± 0.17	9.79^1,a^ ± 0.13	9.89^1,a^ ± 0.05	9.35^1,a^ ± 0.40	9.66^1,a^ ± 0.18	9.85^1,a^ ± 0.08	9.39^1,a^ ± 0.15	9.53^1,a^ ± 0.16	*p* > 0.05
	G_Melox_, *n* = 15	9.26^1,a^ ± 0.21	9.76^1,a^ ± 0.09	9.45^1,a^ ± 0.18	9.44^1,a^ ± 0.16	9.35^1,a^ ± 0.25	8.95^1,a^ ± 0.22	9.29^1,a^ ± 0.20	8.77^1,a^ ± 0.31	7.88^2,b^ ± 0.36	8.82^1,a^ ± 0.32	***p* = 0.006**
	G_CBD_ *n =* 15	8.77^1,a^ ± 0.31	8.96^1,a^ ± 0.24	9.19^1,a^ ± 0.17	9.42^1,a^ ± 0.16	9.41^1,a^ ± 0.18	9.47^1,a^ ± 0.15	9.52^1,a^ ± 0.18	8.40^1,a^ ± 0.29	9.21^1,a^ ± 0.22	9.62^1,a^ ± 0.12	*p* > 0.05
	G_Melox/CBD_ *n =* 15	9.07^1,a^ ± 0.27	9.16^1,a^ ± 0.17	9.39^1,a^ ± 0.15	8.80^1,a^ ± 0.29	9.35^1,a^ ± 0.24	9.25^1,a^ ± 0.15	9.25^1,a^ ± 0.13	9.05^1,a^ ± 0.22	9.44^1,a^ ± 0.18	8.58^1,a^ ± 0.28	*p* > 0.05
	*P* value	*p* > 0.05	*p* > 0.05	*p* > 0.05	*p* > 0.05	*p* > 0.05	*p* > 0.05	*p* > 0.05	*p* > 0.05	***p* = 0.002**	*p* > 0.05	
MIN (mm.)	G_0_ *n =* 15	6.26^1,b^ ± 0.37	7.33^1,a^ ± 0.40	6.74^1,a,b^ ± 0.64	6.72^1,a,b^ ± 0.51	6.63^1,a,b^ ± 0.45	6.99^1,a,b^ ± 0.36	6.90^1,a,b^ ± 0.49	7.99^1,a^ ± 0.34	6.70^1,b^ ± 0.30	6.92^1,a,b^ ± 0.35	***p* = 0.005**
	G_Melox_, *n* = 15	6.75^1,a^ ± 0.30	6.20^1,a^ ± 0.43	6.12^1,a^ ± 0.37	6.34^1,a^ ± 0.35	5.87^1,a^ ± 0.45	6.10^1,a^ ± 0.29	6.02^1,a^ ± 0.34	6.06^2,a^ ± 0.37	5.10^1,a^ ± 0.28	5.84^1,a^ ± 0.35	*p* > 0.05
	G_CBD_ *n =* 15	6.30^1,a^ ± 0.31	6.42^1,a^ ± 0.29	6.32^1,a^ ± 0.19	6.39^1,a^ ± 0.24	6.54^1,a^ ± 0.28	6.46^1,a^ ± 0.20	6.33^1,a^ ± 0.31	5.75^2,a^ ± 0.32	6.09^1,a^ ± 0.30	5.84^1,a^ ± 0.34	*p* > 0.05
	G_Melox/CBD_ *n =* 15	6.55^1,a^ ± 0.33	6.65^1,a^ ± 0.22	6.55^1,a^ ± 0.21	5.75^1,a^ ± 0.26	6.21^1,a^ ± 0.30	6.00^1,a^ ± 0.28	6.09^1,a^ ± 0.21	5.97^2,a^ ± 0.26	6.41^1,a^ ± 0.29	5.79^1,a^ ± 0.27	*p* > 0.05
	*P* value	*p* > 0.05	***p* = 0.049**	*p* > 0.05	*p* > 0.05	*p* > 0.05	*p* > 0.05	*p* > 0.05	***p* = 0.049**	*p* > 0.05	*p* > 0.05	
CH (%)	G_0_ *n =* 15	30.75^1,a^ ± 2.88	30.25^1,a^ ± 2.43	35.71^1,a^ ± 2.40	30.44^1,a^ ± 2.42	31.38^1,a^ ± 1.78	26.44^1,a^ ± 1.62	28.27^1,a^ ± 1.65	28.22^1,a^ ± 2.08	31.44^1,a^ ± 1.90	29.11^1,a^ ± 2.74	*p* > 0.05
	G_Melox_, *n* = 15	26.10^1,a^ 2.24	30.86^1,a^ ± 1.91	32.08^1,a^ ± 2.97	30.79^1,a^ ± 2.14	32.07^1,a^ ± 2.67	32.21^1,a^ ± 2.00	32.90^1,a^ ± 2.22	34.18^1,a^ ± 2.69	30.08^1,a^ ± 1.69	30.08^1,a^ ± 1.98	*p* > 0.05
	G_CBD_ *n =* 15	26.85^1,a^ ± 1.20	30.82^1,a^ ± 1.73	32.29^1,a^ ± 1.56	32.77^1,a^ ± 1.18	33.33^1,a^ ± 1.55	31.93^1,a^ ± 1.53	34.08^1,a^ ± 1.80	33.85^1,a^ ± 2.13	29.20^1,a^ ± 1.78	32.31^1,a^ ± 1.46	*p* > 0.05
	G_Melox/CBD_ *n =* 15	25.50^1,a^ ± 1.29	30.64^1,a^ ± 1.50	29.67^1,a^ ± 1.40	31.46^1,a^ 1.27	31.92^1,a^ ± 2.28	34.64^1,a^ ± 1.67	32.92^1,a^ ± 1.97	34.00^1,a^ ± 1.68	32.36^1,a^ ± 0.88	31.42^1,a^ ± 1.83	*p* > 0.05
	*P* value	*p* > 0.05	*p* > 0.05	*p* > 0.05	*p* > 0.05	*p* > 0.05	*p* > 0.05	*p* > 0.05	*p* > 0.05	*p* > 0.05	*p* > 0.05	
CV (mm./seg.)	G_0_ *n =* 15	3.82^1,a^ ± 0.16	2.98^1,a^ ± 0.29	2.90^1,a^ ± 0.30	2.65^1,a^ ± 0.27	3.11^1,a^ ± 0.25	3.14^1,a^ ± 0.29	3.16^1,a^ ± 0.14	3.18^1,a^ ± 0.24	3.32^1,a^ ± 0.18	3.44^1,a^ ± 0.36	*p* > 0.05
	G_Melox_, *n* = 15	3.16^1,a^ ± 0.16	3.57^1,a^ ± 0.21	3.59^1,a^ ± 0.20	3.14^1,a^ ± 0.17	3.30^1,a^ ± 0.21	3.38^1,a^ ± 0.31	3.27^1,a^ ± 0.28	3.27^1,a^ ± 0.22	3.47^1,a^ ± 0.19	3.28^1,a^ ± 0.26	*p* > 0.05
	G_CBD_ *n =* 15	2.75^1,a^ ± 0.19	3.35^1,a^ ± 0.23	3.44^1,a^ ± 0.20	3.29^1,a^ ± 0.20	3.27^1,a^ ± 0.19	3.61^1,a^ ± 0.19	3.36^1,a^ ± 0.19	3.73^1,a^ ± 0.20	3.22^1,a^ ± 0.18	3.27^1,a^ ± 0.25	*p* > 0.05
	G_Melox/CBD_ *n =* 15	3.43^1,a^ ± 0.21	3.17^1,a^ ± 0.19	3.52^1,a^ ± 0.21	3.28^1,a^ ± 0.18	3.50^1,a^ ± 0.21	3.53^1,a^ ± 0.21	3.35^1,a^ ± 0.12	3.47^1,a^ ± 0.24	3.42^1,a^ ± 0.24	3.37^1,a^ ± 0.19	*p* > 0.05
	*P* value	*p* > 0.05	*p* > 0.05	*p* > 0.05	*p* > 0.05	*p* > 0.05	*p* > 0.05	*p* > 0.05	*p* > 0.05	*p* > 0.05	*p* > 0.05	
MCV (mm./seg.)	G_0_ *n =* 15	5.25^1,a^ ± 0.46	4.61^1,a^ ± 0.54	4.62^1,a^ ± 0.53	4.78^1,a^ ± 0.41	4.75^1,a^ ± 0.44	5.26^1,a^ ± 0.28	5.41^1,a^ ± 0.49	5.51^1,a^ ± 0.56	5.31^1,a^ ± 0.35	5.77^1,a^ ± 0.58	*p* > 0.05
	G_Melox_, *n* = 15	5.16^1,a^ ± 0.24	5.68^1,a^ ± 0.32	5.73^1,a^ ± 0.28	5.84^1,a^ ± 0.46	6.15^1,a^ ± 0.41	6.62^1,a^ ± 0.42	5.93^1,a^ ± 0.41	5.97^1,a^ ± 0.40	5.42^1,a^ ± 0.37	5.46^1,a^ ± 0.41	*p* > 0.05
	G_CBD_ *n =* 15	5.05^1,a^ ± 0.33	5.41^1,a^ ± 0.32	5.89^1,a^ ± 0.38	5.45^1,a^ ± 0.34	5.32^1,a^ ± 0.41	6.29^1,a^ ± 0.35	5.38^1,a^ ± 0.34	5.73^1,a^ ± 0.41	5.10^1,a^ ± 0.25	5.43^1,a^ ± 0.28	*p* > 0.05
	G_Melox/CBD_ *n =* 15	5.22^1,a^ ± 0.32	5.23^1,a^ ± 0.38	6.14^1,a^ ± 0.33	5.43^1,a^ ± 0.32	5.62^1,a^ ± 0.34	5.66^1,a^ ± 0.25	5.69^1,a^ ± 0.27	5.79^1,a^ ± 0.27	5.69^1,a^ ± 0.37	5.90^1,a^ ± 0.30	*p* > 0.05
	*P* value	*p* > 0.05	*p* > 0.05	*p* > 0.05	*p* > 0.05	*p* > 0.05	*p* > 0.05	*p* > 0.05	*p* > 0.05	*p* > 0.05	*p* > 0.05	
Lat. (mm./seg.)	G_0_ *n =* 15	0.19^1,c^ ± 0.00	0.30^1,^ª^,b^ ± 0.02	0.32^1,^ª^,b,1^ ± 0.03	0.33^1,^ª^,b^ ± 0.03	0.27^1,^ª^,b^ ± 0.03	0.26^1,^ª^,b^ ± 0.01	0.27^1,^ª^,b^ ± 0.01	0.27^1,^ª^,b^ ± 0.02	0.22^1,b^ ± 0.01	0.25^1,^ª^,b^ ± 0.01	***p* = 0.002**
	G_Melox_, *n* = 15	0.22^1,a^ ± 0.01	0.25^1,a^ ± 0.01	0.23^2,a^ ± 0.01	0.23^2,a^ ± 0.01	0.23^1,a^ ± 0.01	0.22^1,a^ ± 0.01	0.23^1,a^ ± 0.02	0.22^1,a^ ± 0.01	0.20^1,a^ ± 0.00	0.22^1,a^ ± 0.01	*p* > 0.05
	G_CBD_ *n =* 15	0.23^1,a^ ± 0.01	0.23^1,a^ ± 0.01	0.22^2,a^ ± 0.00	0.26^1,2,a^ ± 0.01	0.22^1,a^ ± 0.02	0.23^1,a^ ± 0.01	0.25^1,a^ ± 0.01	0.21^1,a^ ± 0.02	0.25^1,a^ ± 0.01	0.20^1,a^ ± 0.00	*p* > 0.05
	G_Melox/CBD_ *n =* 15	0.21^1,a^ ± 0.02	0.25^1,a^ ± 0.02	0.23^2,a^ ± 0.01	0.20^2,a^ ± 0.00	0.23^1,a^ ± 0.01	0.23^1,a^ ± 0.01	0.21^1,a^ ± 0.01	0.22^1,a^ ± 0.01	0.23^1,a^ ± 0.01	0.20^1,a^ ± 0.00	*p* > 0.05
	*P* value	*p* > 0.05	*p* > 0.05	***p* = 0.01**	***p* = 0.003**	*p* > 0.05	*p* > 0.05	*p* > 0.05	*p* > 0.05	*p* > 0.05	*p* > 0.05	

^a,b,c^Different literals by row indicate significant differences between events for the same treatment. ^1,2,3,4^Different numerals by column indicate significant differences between treatments for the same event. T = treatments (G_0_: negative group, G_Melox_: Meloxicam group, G_CBD_: Cannabidiol group, G_Melox/CBD_: Mexociam and Cannabidiol group). E: post-surgical events (E_Basal_: 30 min. Pre-surgery; E_30 min._: 30 min post- surgery; E_1h._: 1 h post-surgery; E_2h._: 2 h post-surgery; E_3h._: 3 h post-surgery; E_4h._: 4 h post-surgery; E_8h._: 8 h post-surgery; E_12h._: 12 h post-surgery, E_24h._: 24 h post-surgery; E_48h._: 48 h post-surgery). NPi, Neurological pupil index. Size: Maximum pupil size before constriction. MIN, Pupil diameter at peak constriction; CH, Percentage of pupil change; CV, Constriction velocity; MCV, Maximum constriction velocity; Lat., Latency of constriction. Bold values represent statistically significant differences.

Regarding MIN, dogs in G_0_ registered 6.26 ± 0.37 mm during E_Basal._ This value increased from E_30min_ (7.33 ± 0.40 mm) to 6.92 ± 0.35 mm at E_48h_, having statistically significant differences between events (*p =* 0.005). Differences between treatments were recorded during E_30min_ (*p* = 0.049) and E_12h_ (*p* = 0.049) where G_Melox_, G_CBD_, G_Melox/CBD_ maintained a homogeneous pattern with mean peak constriction values of 5.75 to 6.65 mm. In G_0_, the pupil diameter increased from 0.68–1.13 mm during E_30min_ up to 1.93–2.24 mm during E_12h._

Lat. variable showed values of 0.19 ± 0.00 s in G_0_ animals during E_Basal_; however, this value increased during all postsurgical events, reaching 0.33 ± 0.03 and 0.27 ± 0.02 s at E_2h_ and E_12h_, respectively. Therefore, as observed in the right eye, there was a statistically significant difference between postsurgical events (*p =* 0.002). Moreover, significant differences between treatments at E_1h,_ and E_2h_ were recorded (*p =* 0.01 y *p =* 0.003, respectively), where the Lat. of G_Melox_, G_CBD_, G_Melox/CBD_ was lower than G_0_ during E_1h_ (average of 0.09 s) and E_2h_ (average of 0.1 s).

GCMPS-SF scores are presented in Table 3. In all groups, scores increased from E_Basal_ to the post-operative period (*p =* 0.0001). However, the highest values were recorded in G_0_ during E_30min_, E_1h_, E_2h_, E_3h,_ and E_4h_, in comparison to the rest of the treatments at the same events (*p* = 0.0001). Furthermore, it was found that the pain scores of G_Melox_, G_CBD_, G_Melox/CBD_ did not present differences between groups (*p >* 0.05). Rescue analgesia was administered in one dog included in G_Melox_, G_CBD_, G_Melox/CBD_.

**Table 3:** Pain evaluation scale values (Median ± EE) in the evaluation events (E) of 60 bitches undergoing elective ovariohysterectomy surgeries distributed in 4 study groups: G_0_, G_Melox_, G_CBD_, G_Melox/CBD_.

Parameter	Treatments	Post-surgical Events										P value
		E_Basal_	E_30Min._	E_1h._	E_2h._	E_3h._	E_4h._	E_8h._	E_12h._	E_24h._	E_48h._	
GCMPS-SF	G_0_ *n =* 15	0^1,e^ ± 0	8.00ª^,1^ ± 0.83	9.00^a,1^ ± 0.66	6.00^1,a,b^ ± 0.67	6.00^1,a,b,c^ ± 0.60	4.00^1,b,c,d^ ± 0.51	3.00^1,c,d^ ± 0.49	3.00^1,d^ ± 0.36	3.00^1,c,d^ ± 0.38	3.00^1,d^ ± 0.44	***p* < 0.0001**
	G_Melox_, *n* = 15	0^1,b^ ± 0	3.00^2,a^ ± 0.13	2.50^2,a^ ± 0.54	2.50^2,a^ ± 0.34	2.50^2,a^ ± 0.31	2.00^2,a^ ± 0.27	1.00^1,a^ ± 0.25	1.00^1,a^ ± 0.22	1.00^1,a^ ± 0.22	1.00^1,a^ ± 0.19	***p* < 0.0001**
	G_CBD_ *n =* 15	0^1,d^ ± 0	3.00^2,a^ ± 0.48	3.00^2,a^ ± 0.68	3.00^2,a,b,c^ ± 0.29	3.00^2,a,b^ ± 0.68	3.00^1,2,a,b,c^ ± 0.43	2.00^1,a,b,c^ ± 0.28	3.00^1,a,b,c^ ± 0.38	1.00^1,b,c^ ± 0.23	1.00^1,c^ ± 0.15	***p* < 0.0001**
	G_Melox/CBD_ *n =* 15	0^1,b^ ± 0	3.00^2,a^ ± 0.35	3.00^2,a^ ± 0.53	3.00^2,a^ ± 0.32	3.00^2,a^ ± 0.31	1.50^1,2,a^ ± 0.46	1.00^1,a^ ± 0.27	2.00^1,a^ ± 0.36	1.00^1,a^ ± 0.18	1.00^1,a^ ± 0.27	***p* < 0.0001**
	*P* value	*p* > 0.05	***p* < 0.0001**	***p* < 0.0001**	***p* < 0.0001**	***p* = 0.001**	***p* = 0.03**	*p* > 0.05	*p* > 0.05	*p* > 0.05	*p* > 0.05	

^a,b,c^Different literals by row indicate significant differences between events for the same treatment. ^1,2,3,4^Different numerals by column indicate significant differences between treatments for the same event. T = treatments (G_0_: negative group, G_Melox_: Meloxicam group, G_CBD_: Cannabidiol group, G_Melox/CBD_: Mexociam and Cannabidiol group). E: post-surgical events (E_Basal_: 30 min. Pre-surgery; E_30 min._: 30 min post- surgery; E_1h._: 1 h post-surgery; E_2h._: 2 h post-surgery; E_3h._: 3 h post-surgery; E_4h._: 4 h post-surgery; E_8h._: 8 h post-surgery; E_12h._: 12 h post-surgery, E_24h._: 24 h post-surgery; E_48h._: 48 h post-surgery). GCMPS, Glasgow Composite Pain Score. Bold values represent statistically significant differences.

Finally, in the ROC analysis, it was determined that the Size variable presented a sensitivity of 77.2% and specificity of 96.9% (*p* < 0.0001), while Lat., had a sensitivity of 94.5% and specificity of 88.1% was obtained (*p* < 0.001). Likewise, MIN had a sensitivity of 98.2% and a specificity of 95.6% was recorded (*p* < 0.0001). No significant correlation between the pupillometric indicators was found (Table 4).

**Table 4:** Correlation matrix between Glasgow Composite Pain Scale (GCMPS) values of 60 bitches undergoing elective ovariohysterectomy surgeries distributed in 4 study groups: G_0_, G_Melox_, G_CBD_, G_Melox/CBD_.

Correlation	NPi	Size	MIN	CH	CV	MCV	Lat	GCMPS-SF
Npi	1.00 *p* < 0.0001	0.38 *p* = 1.00	−0.07 *p* = 0.08	0.81 *p* = 1.00	0.00 *p* = 0.90	0.56 *p* = 1.00	−0.11 *p* = 0.81	0.09 *p* = 0.03
Size	0.38 *p* = 0.98	1.00 *p* < 0.0001	0.76 *p* = 1.00	0.10 *p* = 1.00	0.03 *p* = 0.39	0.18 *p* = 1.00	−0.09 *p* = 0.03	0.10 *p* = 0.01
MIN	−0.08 *p* = 0.08	0.76 *p* = 1.00	1.00 *p* < 0.0001	−0.29 *p* = 1.00	0.07 *p* = 0.08	−0,14 *p* = 1.00	−0.07 *p* = 0.08	0.05 *p* = 0.24
CH	0.82 *p* = 0.99	0.10 *p* = 0.01	−0.29 *p* = 1.00	1.00 *p* < 0.0001	0.03 *p* = 0.47	0.50 *p* = 1.00	0.02 *p* = 0.59	0.07 *p* = 0.11
CV	0.01 *p* = 0.98	0.03 *p* = 0.39	0.07 *p* = 0.08	0.03 *p* = 1.00	1.00 *p* < 0.0001	0.03 *p* = 1.00	−0.13 *p* = 0.002	0.03 *p* = 0.39
MCV	0.57 *p* = 0.98	0.18 *p* < 0.0001	−0.14 *p* = 0.001	0.50 *p* = 1.00	0.03 *p* = 0.49	1.00 *p* < 0.0001	−0.06 *p* = 0.14	0.08 *p* = 0.05
Lat	−0.01 *p* = 0.98	−0,09 *p* = 0.02	−0.07 *p* = 0.08	0.02 *p* = 1.00	−0.13 *p* = 0.002	−0.06 *p* = 1.00	1.00 *p* < 0.0001	0.03 *p* = 0.42
GCMPS	0.10 *p* = 0.98	0.10 *p* = 0.01	0.05 *p* = 0.24	0.09 *p* = 1.00	0.03 *p* = 0.39	0.08 *p* = 1.00	0.03 *p* = 0.42	1.00 *p* < 0.0001

GCMPS, Glasgow Composite Pain Score; NPi, Neurological pupil index; Size, Maximum pupil size before constriction; MIN, Pupil diameter at peak constriction; CH, Percentage of pupil change; CV, Constriction velocity; MCV, Maximum constriction velocity; Lat., Latency of constriction.

## 4 Discussion

Among the most significant findings, the pupillometric variables Size, MIN., and Lat. showed higher sensitivity and specificity to identify pain during the postoperative period of dogs undergoing OVH. This suggests that pupillometry is an objective method to recognize acute pain in dogs. The neurophysiological control of the pupil diameter is related to the changes that can be observed in the pupil in animals experiencing pain. Both the sphincter and the dilator muscle control the pupil size. The dilator muscle has sympathetic fibers that increase the pupil diameter or the pupil dilator reflex (27). In humans, pupillometry is currently used to assess pain in pediatrics and traumatology (40–43).

The results indicate that CBD and meloxicam offer equivalent perioperative analgesic quality, without either being superior when these drugs were administered together in the studied animals. Derived from the pupillometric data obtained, it was observed that CBD exhibited similar analgesic activity to meloxicam. This could be explained by the presence of CB1 receptors in neurons of the dorsal horn of the spinal cord (44) and CB2 receptors primarily found in cells of both the immune system and smooth muscle in viscera (45–47). The presence of CB1 and CB2 receptors in the retina, ciliary body, and sympathetic iris fibers has also been suggested (48, 49). Moreover, CBD has a high affinity to CB2 receptors (19).

The analgesic mechanism of action of cannabinoids is mainly by agonism to cannabidiol receptors. The first is the agonism of CB1 receptors, which can induce the activation of Gi/o proteins, inhibiting adenylate cyclase activity and reducing cAMP synthesis. CB1 receptor agonism induces the blockade of voltage-dependent N-type Ca^2+^ channels and an increase in G protein-related K+ channel conductance (19, 50). At the presynaptic level, these actions reduce the release of neurotransmitters such as norepinephrine, histamine, serotonin, dopamine, cholecystokinin, and glutamate in the central nervous system, thereby reducing the perception of nociceptive stimuli (14). Sagar et al. (44) reported that the use of a CB1 receptor agonist decreased Ca^2+^ conductance induced by capsaicin stimulation in dorsal horn neurons of the spinal cord, which could be an explanation for the antinociceptive effect observed in this study.

On the other hand, CB2 agonism could lead to the reduction of an inflammatory response (51) by mediating tumor necrosis factor-alpha (TNF-α) and interleukins from microglia or macrophages (14). Gugliandolo et al. (20) mentioned that the administration of cannabidiol in dogs receiving lipopolysaccharide reduced the presence of interleukin (IL)-10, nuclear factor-kappa B (NF), and the expression of cyclooxygenase 2 (COX-2). Therefore, the reduction in the expression and activity of COX-2 also inhibits the formation of prostaglandins such as prostaglandin E2 (PGE2) and lipoxygenases, subsequently decreasing the expression of proinflammatory metabolites (52). This mechanism of action is also associated with the reduction of proinflammatory cytokine synthesis such as IL-1, IL-8, NFκB, and TNF-α (53, 54). Hence, the evidence suggests that CBD can help to manage or reduce pain by reducing the inflammatory process, possibly being an additional mechanism of pain control.

The pupillometric data obtained in this study showed the analgesic activity of meloxicam due to the preferential inhibition of COX-2 (55, 56). This isoform of COX is the most active during an inflammatory process and is responsible for the production of prostaglandins (57). The inhibition of COX-2 prevents the increase in phospholipase A2 in dorsal horn neurons of the spinal cord, which can consequently prevent the expression of substance P, serotonin, histamine, PGE2, and proinflammatory cytokines (58–60). Preanesthetic administration of meloxicam can prevent peripheral and central sensitization phenomena during nociceptive events due to its pharmacodynamic properties (61, 62).

During the perception of pain, there is an increase in the activity of the sympathetic nervous system (SNS), so NSAIDs like meloxicam can reduce autonomic activity (63, 64). Hernández-Avalos et al. (65) reported that meloxicam increases parasympathetic tone or PTA index similarly to the use of carprofen and paracetamol by decreasing sympathetic nervous system (SNS) activity. The decrease in SNSi activity due to a predominant parasympathetic tone inhibits the stimulation in the Edinger-Westphal nucleus and, in turn, promotes miosis in the pupil (66), as observed in the present results. This effect explains that G_Melox_ obtained the lowest value in the Size variable compared to the other study groups during E_24h_. (*p* < 0.05). However, it should be considered that, in dogs, meloxicam’s half-life is 24 h, which is why re-administration of meloxicam was necessary at this point to maintain adequate plasma levels and therapeutic effect (67), a situation that could have altered the pupillary response of the study subjects.

CBD, by its agonism to CB1 and CB2 receptors, prevents the transmission of nociceptive stimuli by inhibition of central neurotransmitters. On the other hand, meloxicam modulates PGE2 formation (6). Combining both drugs results in a multimodal analgesia that allows pain control at different points of the nociceptive pathway (55, 68). Thus, this could be the possible explanation for G_Melox/CBD_ having a lower MIN compared to the other groups during E_3hr_ (*p* < 0.05) and would reaffirm the fact that CBD exhibits analgesia similar to meloxicam. Therefore, based on our results, CBD can be used to control acute pain in dogs undergoing abdominal surgery and during the immediate postsurgical period. Similarly, according to the findings regarding infrared pupillometry, it can be suggested that the nociceptive response of dogs undergoing OVH and receiving CBD alone or in combination with meloxicam was similar.

Since pain is a subjective condition, its perception may differ among individuals (69). For this reason, it is suggested to use scales that integrate both behavioral and physiological indicators to recognize pain (70–73). In the present research, pain management during the immediate postoperative period could explain the differences observed in this study during the first hours of post-surgery evaluation, since the use of analgesics at the first signs of pain could help control long-term physiological changes and alter the scale scores (74). The scores obtained show the importance of using analgesics before surgery, which could prevent sensitization phenomena and, thus, pain perception (75).

On the other hand, the presence of a larger Size, MIN, and Lat value in G_0_ compared to G_Melox_, G_CBD_, and G_Melox/CBD_ suggests that the pupillary response can be used as a method to recognize postoperative pain in dogs. This has been described in dogs, in whom a positive association between pupil diameter and the value obtained in the numerical rating scale was reported, highlighting that its assessment was limited to the presence or absence of the pupillary reflex (76). The possible neurobiological explanation for the increase in pupil diameter is the increase in SNS activity with catecholamine neurosecretion when animals perceive pain (77). Catecholamines have an effect on α1 adrenergic receptors present in the long ciliary fibers of the iris dilator muscle, which activation would lead to pupil dilation (78, 79). This was observed in G_0_ animals during E_2hr_, values that were also associated with increasing scores in the GCMPS-SF. A similar association between pupil diameter and pain scales has been reported in human medicine (43, 80, 81). Therefore, the present findings suggest a possible relationship between pain scales and the pupillary response in animals. Although further research is needed to establish the correlation between both methods to evaluate pain, the application of pupillometry could help to refine pain assessment in companion animals (4, 8).

Size and MIN represent an increase in the pupil diameter; however, the response to the light stimulus increased both in the left and right eye. This can also be evaluated through Lat, where the highest values were recorded in G_0_ in both the left and right eyes, in comparison with G_Melox_, G_CBD_, and G_Melox/CBD._ This indicates that the pupil speed is greater when faced with a light stimulus (27). In this sense, Mills et al. (28) suggested that the maximum value of Lat in dogs is 0.30 s, a value that was below the ones reported in the present study, possibly due to nociception. The pupillary response observed in animals during the perception of pain is related to the activation of the Locus Coeruleus, a region that contains pre-motor and excitatory sympathetic neurons that are projected to preganglionic neurons in the Edinger-Westphal nucleus and present in α2 adrenergic receptors. Through sympathoexcitation and parasympathetic inhibition, these fibers cause pupillary dilation, decreasing the response to light (82). Therefore, this could be the first time addressing the influence that these drugs have on the pupil diameter of dogs.

The increase in these values occurred at E_30min_, E_1h_, and E_2h_, when animals in G_0_ received rescue analgesia. In this sense, although there could be a residual effect of anesthetics, it is reported that pupil dilation has a positive relation with anesthetic depth (83, 84). This effect could only be observed in G_0_ at E_30min_, in contrast to the G_Melox_, G_CBD_, and G_Melox/CBD_ groups, which was not observed at E_1h_. and E_2h_. It is necessary to mention that meloxicam has an elimination half-life of 24 h in dogs (67), while CBD has an elimination half-life of 3 to 5 days (14). This coincides with the increase in pain scores assessed with the behavioral-based scale in G_0_.

The present findings suggest that pupillometry could be used to recognize pain in dogs subjected to OVH. However, it is necessary to consider that increased values during the immediate postsurgical period where pain control is essential to avoid the physiological consequences of pain might coincide with these critical events (72, 73). This would explain why the pupillometric parameters and pain scores decreased in the subsequent events. Dyson (85) explains that pain control during the first hours after surgery reduces the risk of short- and long-term complications. Therefore, this evidence could lead to corroborating the theory that this tool can be used as an objective and quantitative way of acute pain in animals (86). Additionally, the sensitivity and specificity for MIN and Lat. were greater than 80%, possibly making it a reliable tool for assessing pain in animals. This has been reported in humans, recording a sensitivity of around 100% and a specificity of 77% (80). Regardless of the species, future studies need to consider the clinical application of pupillometry.

Regarding rescue analgesia, it was observed that G_Melox_ and G_CBD_ required more rescue analgesia than G_Melox/CBD_ (G_Melox_ = 1, G_CBD_ = 1, G_Melox/CBD_ = 0). This is due to the effect of multimodal analgesia in which CBD inhibits the nociceptive stimulus while meloxicam negatively alters the nociceptive signal at the peripheral level, preventing pain perception (68, 87). However, when comparing the number of animals that required rescue analgesia in G_Melox_ and G_CBD_, these were significantly lower than G_0_, where all animals received rescue analgesia due to the lack of an analgesic protocol before the surgery. Thus, these observations add to the importance of providing analgesia to dogs before the surgical procedure to avoid pain-related complications during the postoperative period (88). Furthermore, at E_30min_ the increase in pupillometry parameters was related to an increase in the GCMPS score. However, this might be attributed to the residual effect of general anesthetics and sedatives such as α2 agonists (89). Thus, this could be considered a limitation on the use of pupillometry in surgical patients.

One of the main limitations of the present study is that current pupillometry does not consider the anatomical and conformational characteristics of a dog’s eyes. For example, the iris pigmentation and morphology might affect the accuracy of pupillometric variables (90). This needs to be established in future research when implementing pupillometry as a complementary tool to assess pain. Another field of research would be implementing pupillometry during other surgical procedures such as trauma surgery where there is a greater risk of pain perception. Other limitation could be the level of fear that awake animals might experience, which needs further study to improve the application of pupillometry in veterinary medicine. Likewise, physiological parameters are not reported during the postoperative period, which can be modified due to the painful experience. This limitation arises from the incorporation of these parameters into another paper derived from the present research. Finally, another important perspective is the correlation with other methods that have been suggested to evaluate pain, such as the physiological parameters, the parasympathetic tone index monitor and infrared thermography (90–98).

## 5 Conclusion

According to the results obtained through pupillometry and the GCMPS-SF scores, CBD alone or in combination with meloxicam has a similar analgesic effect for the control of acute pain in dogs. The findings of the present study suggest that infrared pupillometry could be implemented as a tool to recognize acute pain in ovariohysterectomized bitches.

## Data availability statement

The original contributions presented in the study are included in the article/Supplementary material, further inquiries can be directed to the corresponding author/s.

## Ethics statement

The animal studies were approved by the Ph.D. Program in the Biological and Health Science Academic Committee (number CBS.066.21). The studies were conducted in accordance with the local legislation and institutional requirements. Written informed consent was obtained from the owners for the participation of their animals in this study.

## Author contributions

AC-A: Writing – review & editing, Writing – original draft, Supervision, Methodology, Formal analysis. JM-B: Writing – review & editing, Writing – original draft, Supervision. IH-Á: Writing – review & editing, Writing – original draft, Supervision, Methodology, Conceptualization. PM-M: Writing – review & editing, Writing – original draft, Supervision, Methodology. AM-C: Writing – review & editing, Writing – original draft, Supervision. AD-O: Writing – review & editing, Writing – original draft, Supervision. DM-R: Writing – review & editing, Writing – original draft, Supervision, Formal analysis, Conceptualization.
