# Supplementary material for: Assessment of the nociceptive response to the use of cannabidiol alone and in combination with meloxicam through infrared pupillometry in female dogs undergoing elective ovariohysterectomy
Source: Front Vet Sci. 2024 Jul 4;11:1380022. doi: 10.3389/fvets.2024.1380022 (PMC11256235; doi:10.3389/fvets.2024.1380022)
Supplement: Supplementary file 1 [file Data_Sheet_1.PDF]

Consultorio Médico Veterinario

Can & Cat

Calle 5 No. 368 Colonia Profesor Cristóbal Higuera. Atizapán de Zaragoza. Edo. de México. C.P. 52940.

Horarios:
Lunes a sábado de 11:00 am a 7:00 pm

Teléfonos: 50772101

Emergencias:
0445539401962
0445528654895

Consentimiento y autorización de cirugía

Atizapán de Zaragoza, Estado de México a_______de__________de 20_____

Al firmar esta forma yo (nombre)____certifico que soy el dueño legal o persona autorizada por el propietario del paciente, descrito en la siguiente reseña:

**Identificación:**	**Especie:**
**Raza:**	**Sexo:**
**Edad y peso:**	**Color:**

Así por este conducto, doy consentimiento al Consultorio Médico Veterinario Can & Cat para que mi mascota sea intervenida bajo el siguiente procedimiento quirúrgico: ________________________________ _________________________, mismo que será realizado con el protocolo anestésico que me ha sido propuesto.

Así mismo, se me ha informado que de acuerdo a la clasificación ASA (Sociedad Americana de Anestesiólogos) para estimar el riesgo anestésico, la salud de mi mascota se encuentra clasificada como:

**Clasificación (Marque con una X)**		**Descripción**
	**I**	Pacientes sanos sometidos a cirugía electiva.
	**II**	Pacientes que presentan enfermedad pre—existente de ligera a moderada, no involucrando cavidad torácica, abdominal y/o pélvica.
	**III**	Pacientes que presentan enfermedades sistémicas, en las cuales se encuentran involucradas las cavidades torácica, abdominal y/o pélvica.
	**IV**	Pacientes con enfermedades pre—existentes y alteraciones sistémicas severas, que se deben de corregir antes de la anestesia .
	**V**	Pacientes moribundos que pueden fallecer en menos de 24 horas, ya sea que se practique o no la cirugía.
	**U**	Paciente que debe ingresar a cirugía de urgencia, ya que su condición de salud así lo requiere.

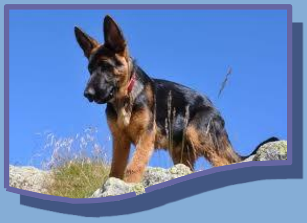

DEDICADOS AL CUIDADO Y BIENESTAR DE SU MASCOTA

El costo de la intervención quirúrgica será de $___________________MN mismo que me ha sido informado y he aceptado en los términos ya señalados. Los honorarios citados cubrirán lo referente a cirugía, anestésicos, medicamentos y retiro de sutura. El monto total (100%) del costo de la cirugía deberá ser cubierto antes de que se realice el procedimiento. Del mismo modo, acepto que los cuidados post operatorios son mi responsabilidad (propietario), ya que el paciente estará bajo mi cuidado en mi domicilio.

Consultorio Médico Veterinario

Can & Cat

Calle 5 No. 368 Colonia Profesor Cristóbal Higuera. Atizapán de Zaragoza. Edo. de México. C.P. 52940.

Horarios:
Lunes a sábado de 11:00 am a 7:00 pm

Teléfonos: 50772101

Emergencias:
0445539401962
0445528654895

Consentimiento y autorización de cirugía

Atizapán de Zaragoza, Estado de México a_______de__________de 20_____

Por otra parte, he leído y aceptado la información indicada en el anverso de este consentimiento, el cual se encuentra basado en el numeral 10.1.1 de la NOM-168-SSA1-1998 (cabe destacar que este precepto legal aplica a medicina humana pero que es tomado como base y se encuentra referida en la elaboración del presente consentimiento).

También he realizado las preguntas oportunas y he sido informado de manera clara, correcta y amablemente de las ventajas y/o riesgos que representa el procedimiento anestésico—quirúrgico, por lo cual libero de toda responsabilidad civil y/o legal al personal médico que labora en el Consultorio Médico Veterinario Can & Cat.

Del mismo modo, acepto las modificaciones de los métodos que se puedan producir en el transcurso de dichos procedimientos y que se justifique por una mejora de la calidad de los mismos y en beneficio de la salud del paciente, aunque ello pueda suponer un incremento en el costo del procedimiento.

_________________________________ _________________________________

Nombre y firma del propietario y/o representante

Nombre y firma del testigo presencial

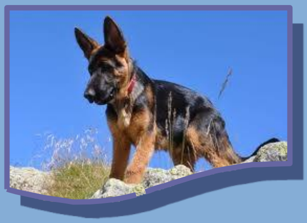

Atentamente

DEDICADOS AL CUIDADO Y BIENESTAR DE SU MASCOTA

PhD. Ismael Hernández Ávalos
Ced. Profesional 3920116

M en MVZ Agatha Elisa Miranda Cortés
Ced. Profesional 5190964
